# Complete remission of metastatic pheochromocytoma in ^123^I-metaiodobenzylguanidine scintigraphy after a single session of ^131^I-metaiodobenzylguanidine therapy: a case report

**DOI:** 10.1186/s13104-017-3095-6

**Published:** 2017-12-19

**Authors:** Teruaki Sugino, Ryosuke Ando, Rei Unno, Keitaro Iida, Taku Naiki, Shuzo Hamamoto, Kentaro Mizuno, Atsushi Okada, Yukihiro Umemoto, Noriyasu Kawai, Keiichi Tozawa, Yutaro Hayashi, Anri Inaki, Daiki Kayano, Seigo Kinuya, Takahiro Yasui

**Affiliations:** 1 Department of Nephro-urology, Nagoya City University Graduate School of Medical Sciences, 1, Kawasumi, Mizuho-cho, Mizuho-ku, Nagoya, 467-8601 Japan; 2 Department of Nuclear Medicine, Faculty of Medicine, Institute of Medical, Pharmaceutical and Health Sciences, Kanazawa University, Kanazawa, Japan

**Keywords:** Adrenal gland neoplasm, Neuroendocrine tumor, Malignant pheochromocytoma, Germ-line mutation, ^131^I-metaiodobenzylguanidine

## Abstract

**Background:**

Pheochromocytomas are rare neuroendocrine tumors, with a malignancy frequency of approximately 10%. The treatment of malignant pheochromocytoma is palliative, and the traditional management strategy has limited efficacy. Furthermore, no clear criteria exist for the treatment of metastatic pheochromocytoma, especially for unresectable lesions. We report a case of complete remission of metastatic pheochromocytoma in ^123^I-metaiodobenzylguanidine (MIBG) scintigraphy after a single session of ^131^I-MIBG therapy.

**Case presentation:**

A 61-year-old woman had a right adrenal grand tumor and lymph node metastasis on the hilum of the right kidney, both of which incorporated MIBG. After surgery, immunostaining of a tumor specimen showed expression of the tumor makers chromogranin and synaptophysin. One year postoperatively, abdominal computed tomography revealed a local recurrence and retroperitoneal lymph node swelling. The local recurrence was positive for MIBG uptake, whereas the swollen retroperitoneal lymph nodes were negative. She underwent surgery again, but the local recurrence was unresectable because of rigid adhesion to the surrounding tissue. Immunostaining of an intraoperatively extracted swollen retroperitoneal lymph node showed expression of tumor markers. The patient then underwent a single session of ^131^I-MIBG therapy (7.4 GBq, 200 mCi), after which the residual lesions no longer incorporated MIBG, and a complete response in ^123^I- metaiodobenzylguanidine (MIBG) scintigraphy was achieved. The ^131^I-MIBG treatment was repeated 6 months later. None of the lesions were positive for MIBG uptake.

**Conclusions:**

^131^I-MIBG therapy efficaciously treats unresectable lesions that are positive for MIBG uptake.

## Background

Pheochromocytomas (PCCs) are rare neuroendocrine tumors with an incidence of 0.4–9.5/1,000,000 individuals [1]. Approximately 10% of PCCs are malignant [2]. Evaluating PCC malignancy based on pathogenic characteristics is difficult; one criterion is metastasis or recurrence in a tissue other than chromaffin tissue [3].

Most PCCs are sporadic, but some are hereditary [4]. It has recently been reported that PCC is associated with germline succinate dehydrogenase B (*SDHB*) mutations, which are found in up to 50% of patients with malignant PCC [5].

The treatment of malignant PCC remains controversial because there are very few cases of PCC. ^131^I-metaiodobenzylguanidine (MIBG) therapy is the most studied targeted radiotherapy in PCC patients. The purpose of this report is to present a case of PCC and to discuss the treatment of malignant PCC.

## Case presentation

**Fig. 1:**
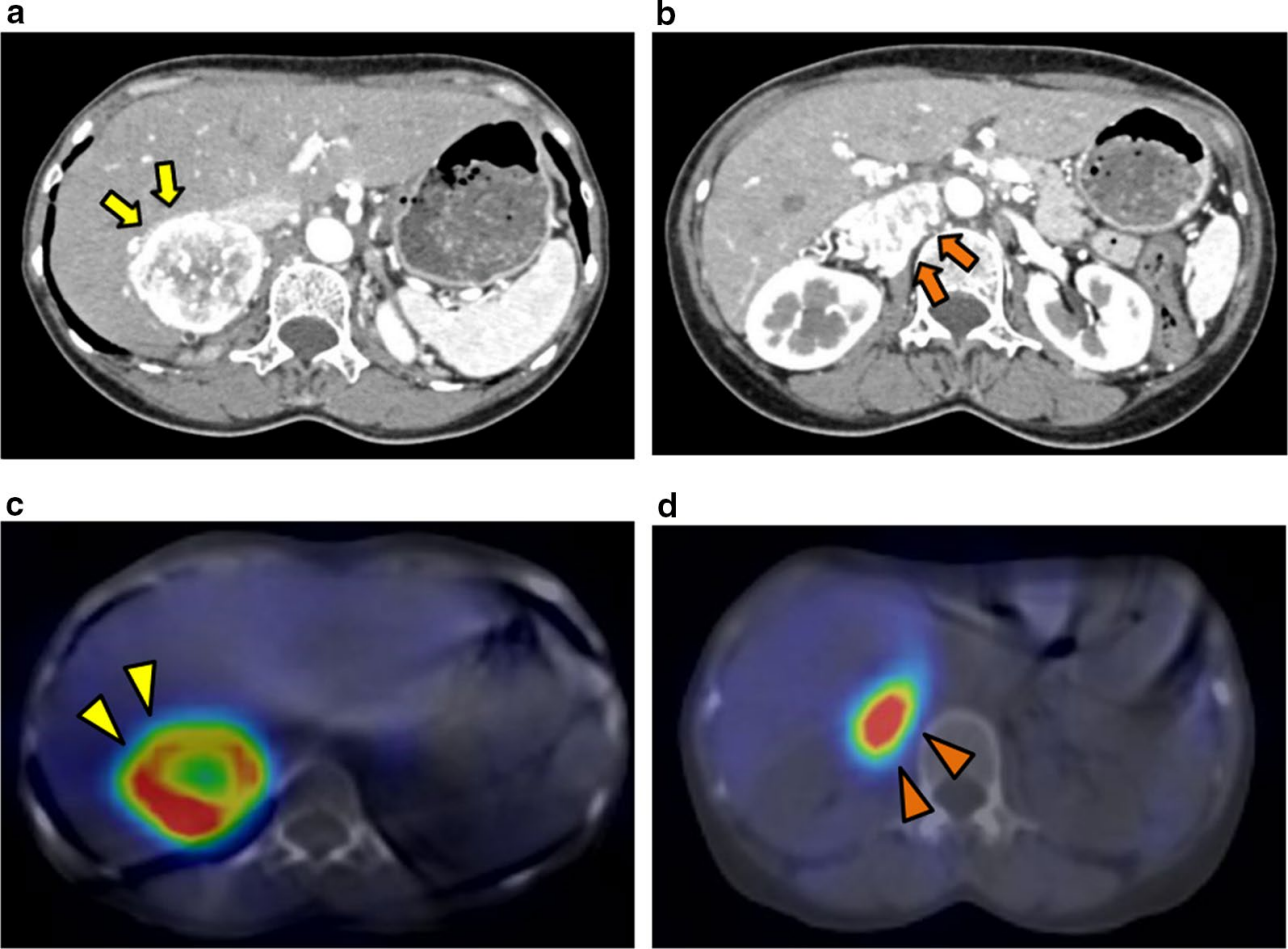
Abdominal computed tomography at the first visit shows the right adrenal gland tumor (**a**, yellow arrows) and the lymph node metastasis (**b**, orange arrows) on the hilum of the right kidney. ^123^I-metaiodobenzylguanidine (MIBG) scintigraphy shows MIBG in the adrenal tumor (**c**, yellow arrowheads) and lymph node metastasis (**d**, orange arrowheads)

A 61-year-old woman (height, 148.9 cm; body mass index, 21.3 kg/m^2^) had a right adrenal gland tumor. She had no relevant medical history or family history. Endocrinological examination of her blood indicated a high noradrenaline level (3429 pg/mL). The 24-h urine collection revealed high levels of noradrenaline (728 µg/day) and dopamine (1092 µg/day). On admission, her blood pressure was 169/85 mmHg. Abdominal computed tomography (CT) revealed a 5-cm right adrenal gland tumor and a 4-cm lymph node metastasis on the hilum of the right kidney. ^123^I-MIBG scintigraphy revealed that both were positive for MIBG uptake (Fig. 1). The patient was diagnosed with malignant PCC.

**Fig. 2:**
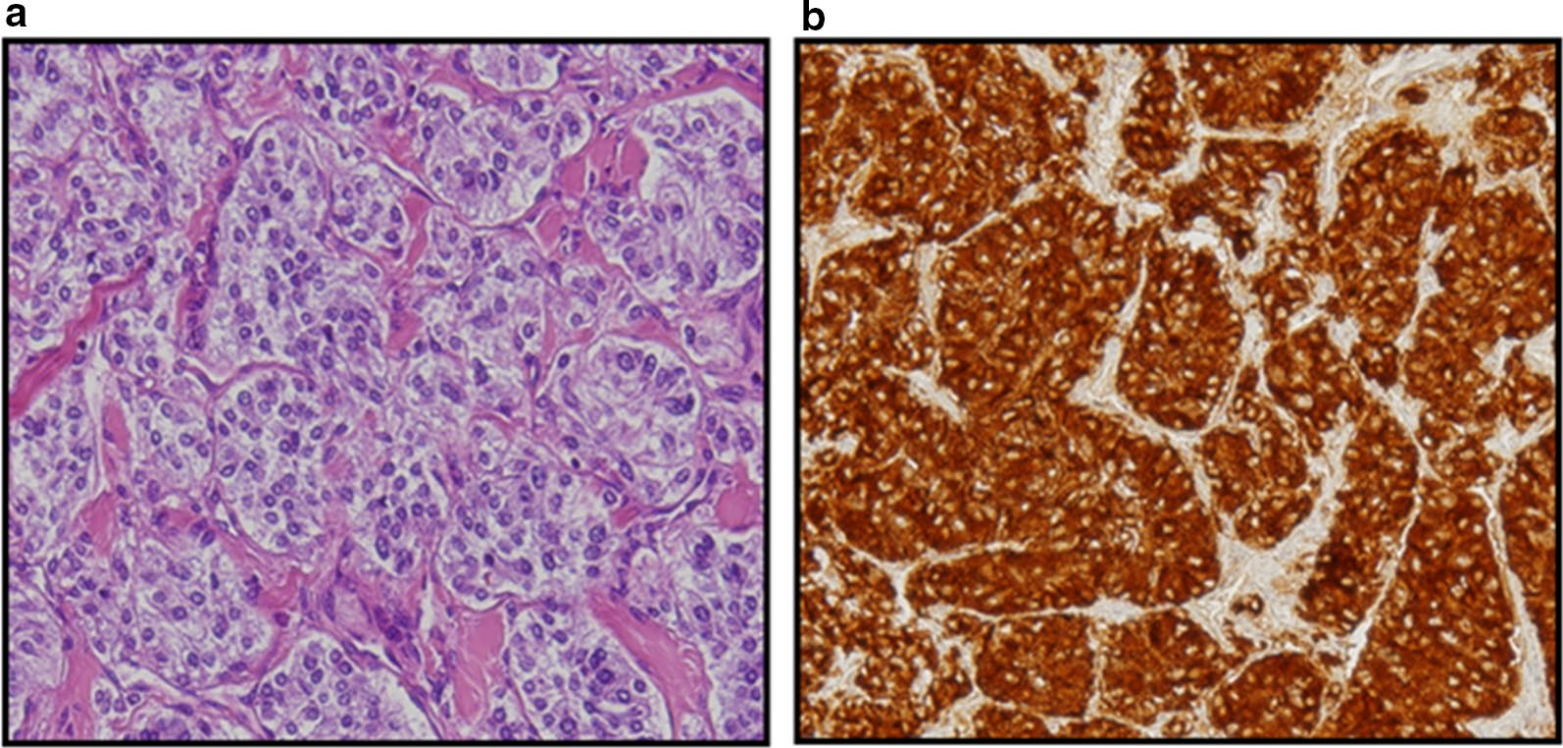
**a** Hematoxylin and eosin staining shows that the tumor has a Zellballen pattern (magnification, ×40). **b** Immunostaining of a tumor sample for chromogranin (magnification, ×40)

The right adrenal tumor and lymph node metastasis were resected. Before the operation, she took oral doxazosin mesylate. After the operation, her blood pressure and serum catecholamine levels immediately normalized. Hematoxylin and eosin staining of a tumor specimen indicated that the tumor had a Zellballen pattern. Immunostaining showed expression of chromogranin and synaptophysin (Fig. 2), which is helpful in the pathological diagnosis of PCC, but not SDHB.

Twelve months postoperatively, abdominal CT revealed a 1.0-cm mass in the area where the primary tumor had existed. It also revealed some swollen retroperitoneal lymph nodes that were less than 1.0 cm. The mass in the area where the primary tumor had existed was positive for MIBG uptake, whereas the swollen retroperitoneal lymph nodes were negative.

**Fig. 3:**
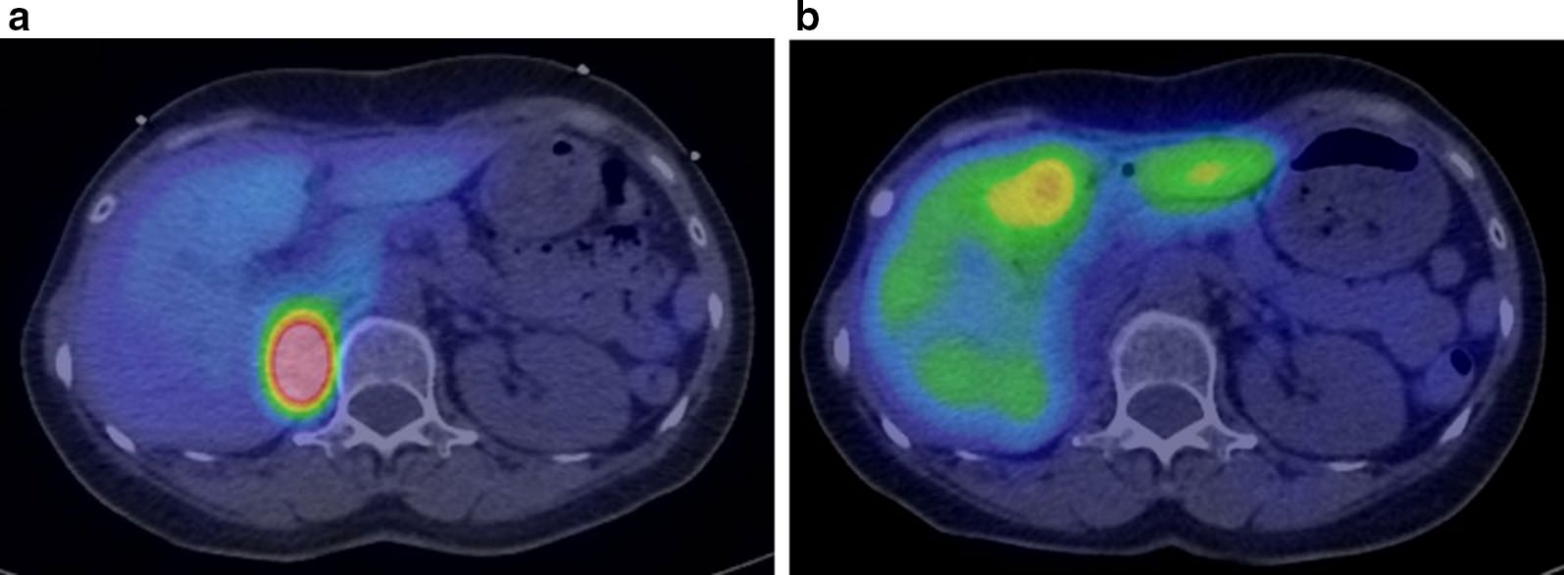
^123^I-Metaiodobenzylguanidine (MIBG) uptake before (**a**) and after (**b**) ^131^I-MIBG treatment. After a single treatment, the lesions no longer contained ^123^I-MIBG (**b**)

The patient underwent another surgery. The local recurrence was rigidly adhered to the surrounding tissue and therefore was impossible to extract. We intraoperatively extracted a swollen lymph node in the retroperitoneal area for sampling. Immunostaining revealed tumor cells in the lymph node. After the operation, the unresectable local recurrence gradually enlarged. Twelve months later at another hospital, she was treated with ^131^I-MIBG at a dose of 7.4 GBq (200 mCi). After one treatment, the residual lesions no longer incorporated MIBG (Fig. 3). She underwent a second ^131^I-MIBG treatment 6 months after the first. She experienced no adverse event over grade 3 (Common Terminology Criteria for Adverse Events v4.0). To date (1 month has passed), no lesions have been positive for MIBG uptake.

## Discussion and conclusions

Although there is no curative management for malignant PCC, our case report suggests that ^131^I-MIBG may be an effective treatment for some patients. Current therapeutic targets for malignant PCC are to suppress the excessive secretion of catecholamines, to extend progression-free survival and to prevent cardiac insufficiency, which is the most common cause of death in patients with malignant PCC [6].

The first-line treatment for malignant PCC is surgery. Complete resection can provide catecholamine control and allow long-term remission. Furthermore, debulking surgery may increase the efficacy of subsequent treatments. The treatment for unresectable lesions without MIBG uptake is chemotherapy. The most common chemotherapy regimen is the combination of cyclophosphamide, vincristine, and dacarbazine (i.e., the CVD regimen). The treatment for unresectable lesions with MIBG uptake is ^131^I-MIBG therapy or chemotherapy. However, no clear criteria exist with regard to which treatment is better.

For patients with malignant PCC, the median progression-free survival time is 24–36 months after ^131^I-MIBG treatment [7, 8] and 20–40 months after chemotherapy [9, 10]. There has been no prospective study compared both treatments. In a previous report, ^131^I-MIBG was effective for patients who had small tumors or no bone metastasis [11]. Because our patient fulfilled these criteria, a single ^131^I-MIBG treatment (7.4 GBq, 200 mCi) resulted in significant effectiveness.

Approximately 35% of PCC patients have hereditary PCC [4]. Germline mutations associated with malignancy include *SDHB* mutations. A recent report suggests that immunostaining of *SDHB* is a promising marker which indicates the presence of an *SDHB* mutation [12], as was observed in our case. Because tumors with germline mutations in the *SDHB* gene are characterized by angiogenesis [13], angiogenetic therapy (e.g., administration of a tyrosine kinase inhibitor) may effectively treat patients with such mutations. This perhaps includes our patient, who may have an *SDHB* mutation.

Herein, we described successful ^131^I-MIBG treatment of an unresectable malignant PCC. ^131^I-MIBG administration could be an efficacious means of treating patients with malignant PCCs that are positive for MIBG uptake.

## Abbreviations

CT: computed tomography
MIBG: metaiodobenzylguanidine
PCC: pheochromocytoma
SDHB: succinate dehydrogenase B

## References

[1] Adler JT, Meyer-Rochow GY, Chen H, Benn DE, Robinson BG, Sippel RS (2008). Pheochromocytoma: current approaches and future directions. Oncologist.

[2] Chrisoulidou A, Kaltsas G, Ilias I, Grossman AB (2007). The diagnosis and management of malignant phaeochromocytoma and paraganglioma. Endocr Relat Cancer.

[3] Parenti G, Zampetti B, Rapizzi E, Ercolino T, Giachè V, Mannelli M (2012). Updated and new perspectives on diagnosis, prognosis, and therapy of malignant pheochromocytoma/paraganglioma. J Oncol.

[4] Pacak K, Eisenhofer G, Ahlman H, Bornstein SR, Gimenez-Roqueplo AP, Grossman AB (2007). International symposium on pheochromocytoma. pheochromocytoma: recommendations for clinical practice from the first international symposium. October 2005. Nat Clin Pract Endocrinol Metab.

[5] Ayala-Ramirez M, Feng L, Johnson MM, Ejaz S, Habra MA, Rich T (2011). Clinical risk factors for malignancy and overall survival in patients with pheochromocytomas and sympathetic paragangliomas: primary tumor size and primary tumor location as prognostic indicators. J Clin Endocrinol Metab.

[6] Baudin E, Habra MA, Deschamps F, Cote G, Dumont F, Cabanillas M (2014). Therapy of endocrine disease: treatment of malignant pheochromocytoma and paraganglioma. Eur J Endocrinol.

[7] Krempf M, Lumbroso J, Mornex R, Brendel AJ, Wemeau JL, Delisle MJ (1991). Use of m-[131I]iodobenzylguanidine in the treatment of malignant pheochromocytoma. J Clin Endocrinol Metab.

[8] Gedik GK, Hoefnagel CA, Bais E, Olmos RA (2008). ^131^I-MIBG therapy in metastatic phaeochromocytoma and paraganglioma. Eur J Nucl Med Mol Imaging.

[9] Huang H, Abraham J, Hung E, Averbuch S, Merino M, Steinberg SM (2008). Treatment of malignant pheochromocytoma/paraganglioma with cyclophosphamide, vincristine, and dacarbazine: recommendation from a 22-year follow-up of 18 patients. Cancer.

[10] Tanabe A, Naruse M, Nomura K, Tsuiki M, Tsumagari A, Ichihara A (2013). Combination chemotherapy with cyclophosphamide, vincristine, and dacarbazine in patients with malignant pheochromocytoma and paraganglioma. Horm Cancer.

[11] Yoshinaga K, Oriuchi N, Wakabayashi H, Tomiyama Y, Jinguji M, Higuchi T (2014). Drafting committee for guidelines on internal radiotherapy with ^131^I-MIBG; Japanese Society of Nuclear Medicine in Oncology and Immunology; Japanese Society of Nuclear Medicine. Effects and safety of ^131^I-metaiodobenzylguanidine (MIBG) radiotherapy in malignant neuroendocrine tumors: results from a multicenter observational registry. Endocr J.

[12] Pai R, Manipadam MT, Singh P, Ebenazer A, Samuel P, Rajaratnam S (2014). Usefulness of succinate dehydrogenase B (SDHB) immunohistochemistry in guiding mutational screening among patients with pheochromocytoma-paraganglioma syndromes. APMIS.

[13] Favier J, Igaz P, Burnichon N, Amar L, Libé R, Badoual C (2012). Rationale for anti-angiogenic therapy in pheochromocytoma and paraganglioma. Endocr Pathol.

